# Differential regional perfusion of the human anterior cruciate ligament: quantitative magnetic resonance imaging assessment

**DOI:** 10.1186/s40634-022-00486-8

**Published:** 2022-05-30

**Authors:** Kenneth M. Lin, Harmen D. Vermeijden, Craig E. Klinger, Lionel E. Lazaro, Scott A. Rodeo, Jonathan P. Dyke, David L. Helfet, Gregory S. DiFelice

**Affiliations:** 1 Department of Orthopaedic Surgery, Hospital for Special Surgery, 535 East 70th Street, New York, NY 10021 USA; 2 Miami Orthopedics and Sports Medicine Institute, Baptist Health South Florida, Miami, FL USA; 3 Citigroup Biomedical Imaging Center and Weill Cornell Medicine, New York, NY USA

**Keywords:** ACL vascularity, Perfusion, Quantitative MRI, ACL primary repair, ACL reconstruction, Remnant preservation

## Abstract

**Purpose:**

Surgical reconstruction is the current standard for ACL rupture treatment in active individuals. Recently, there is renewed interest in primary repair of proximal ACL tears. Despite this, ACL biology and healing potential are currently not well understood. Vascularity is paramount in ACL healing; however, previous ACL vascularity studies have been limited to qualitative histological and dissection-based techniques. The study objective was to use contrast-enhanced quantitative-MRI to compare relative perfusion of proximal, middle, and distal thirds of the in situ ACL. We hypothesized perfusion would be greatest in the proximal third.

**Methods:**

Fourteen cadaveric knees were studied (8 females, 6 males), age 25–61 years. Superficial femoral, anterior tibial, and posterior tibial arteries were cannulated; without intraarticular dissection. Contrast-enhanced quantitative-MRI was performed using a previously established protocol. ACL regions corresponding to proximal, middle, and distal thirds were identified on sagittal-oblique pre-contrast images. Signal enhancement (normalized to tibial plateau cartilage) was quantified to represent regional perfusion as a percentage of total ACL perfusion. Comparative statistics were computed using repeated measures ANOVA, and pairwise comparisons performed using the Bonferroni method.

**Results:**

Relative perfusion to proximal, middle, and distal ACL zones were 56.0% ±17.4%, 28.2% ±14.6%, and 15.8% ±16.3%, respectively (*p* = 0.002). Relative perfusion to the proximal third was significantly greater than middle (*p* = 0.007) and distal (*p* = 0.001). No statistically relevant difference in relative perfusion was found to middle and distal thirds (*p* = 0.281). Post-hoc subgroup analysis demonstrated greater proximal perfusion in males (66.9% ± 17.3%) than females (47.8% ± 13.0%), *p* = 0.036.

**Conclusion:**

Using quantitative-MRI, in situ adult ACL demonstrated greatest relative perfusion to the proximal third, nearly 2 times greater than the middle third and 3 times greater than the distal third. Knowledge of differential ACL vascular supply is important for understanding pathogenesis of ACL injury and the process of biological healing following various forms of surgical treatment.

## Background

Anterior cruciate ligament (ACL) injury is common in the active population, with incidence of 75 per 100,000 person-years [1]. Surgical intervention often is recommended to restore knee stability, prevent further injury, and enable safe return to sport or activity [2–5]. The ACL traditionally was considered to have poor intrinsic healing capacity [6], as early studies of repair, irrespective of tear location, showed poor outcomes [7, 8]. The surgical standard for complete ACL rupture is ligament reconstruction. However, a subpopulation of patients with ACL rupture have been found to perform well following nonoperative management [9]. Recent histological study suggested that proximal one-third ACL tears may have intrinsic healing response similar to that of the medial collateral ligament (MCL) [10]. Along this line of thought, there has been renewed interest in primary repair of proximal ACL tears [11], although data remains limited [12, 13]. Regardless of treatment strategy, with continued scientific advancement, it is important to understand ACL biology and its pathophysiologic response to injury.

A key component of the healing cascade is vascularity, specifically angiogenic response to injury and surgical treatment, as vascular influx is the first healing stage. The ACL is perfused by the middle geniculate artery [14], and perfusion is thought greatest at the terminal ends of the ligament [15]. The ACL is nearly entirely supplied by branches traveling through the synovium, rather than the bony insertion [16, 17]. Prior research has also found that there is a higher number of blood vessels near the proximal portion of the ACL [17]. From a healing standpoint, animal studies have shown biologic augmentation using stromal cells and various growth factors can improve ACL graft-tunnel healing [18–20].

While it is known vascularity is integral to ACL healing, previous knowledge has been gained from histological dissection studies or animal models. For greater direct clinical relevance, vascularity should be studied in situ in human tissues using methods more closely resembling the clinical setting. Knowledge of relative perfusion using noninvasive imaging could be applied to assessment of patients in the injury setting, as well as evaluation of ligament/graft perfusion and healing post-operatively. A more precise understanding of ACL vascularity not only improves the basic science knowledge of ACL biology, but may also carry implications for ACL surgery indications. To date, there are no studies in intact human knees, using standard clinically available imaging modalities, that quantitatively assessed ACL vascularity.

The study objective was to quantify the in situ relative vascularity of the adult human ACL by anatomic zone using gadolinium-enhanced MRI. We hypothesized perfusion would be greatest in the proximal third of the ACL.

## Methods

### Specimens

Following institutional review board approval, 14 unmatched human cadaveric knee specimens were acquired (fresh-frozen, mid-femur to mid-tibia; Anatomy Gifts Registry, Hanover, Maryland). All specimens were pre-screened excluding any with associated pathology, injury or prior knee surgery. There were 8 females and 6 males (average age 51.1 years; SD 9.4, range 25–61). Causes of death were suicide (1), neurodegenerative disorder (1), heart failure (1), drug overdose (1), malabsorption syndrome due to gastric bypass (1), multiple sclerosis (1), malignant neoplasm (1), pancreatic cancer (1), lung cancer (1), metastatic renal cancer (1), lymphoma (1), kidney failure (1), throat cancer (1), metastatic thyroid cancer (1). Dissection was performed at our anatomy laboratory and MRI at our academic affiliate, (Simulation Learning and Training Center at the Hospital for Special Surgery, and Citigroup Biomedical Imaging Center of Weill Cornell Medicine, respectively).

### Cannulation procedure

Arterial cannulas (DLP 30000; Medtronic, Minneapolis, MI) were inserted and sutured in place using 2–0 Vicryl sutures (Johnson & Johnson, New Brunswick, NJ) into the superficial femoral artery (SFA; proximal to branch-point of the supreme genicular artery), the anterior tibialis artery (ATA; distal to branch-point of the anterior recurrent artery); and the posterior tibialis artery (PTA; distal to branch-point of the anterior tibialis artery and proximal to the bifurcation of the peroneal and posterior tibialis arteries). The arterial dissection and cannulation procedure of the current study was the same technique as detailed in a prior cadaveric study by Lazaro et al. [21], and similar techniques were used for the contrast MRI protocol to prior quantitative-MRI research [22–24].

### Quantitative MRI procedure

MRI scans were performed using a 3.0-T Excite General Electric MRI scanner (GE Healthcare, Milwaukee, Wisconsin) with a quadrature knee coil. The quantitative-MRI (qMRI) protocol consisted of a 20 cm field of view, 512 × 384 matrix size, with 2 mm slice thickness, and was utilized pre- and post-contrast infusion using GE LAVA MRI sequences for pre- and post-contrast imaging allowing improved image detail of signal enhancement. The image acquisition parameters consisted of repetition and echo times of 18.6 and 5.3 ms, respectively, and a flip angle of 35°. An image resolution of 0.4 · 0.4 · 1.0 mm was produced following MRI image reconstruction. Intravenous (IV) tubing was connected to each arterial cannula (Topspins Inc., Extension Set 001.4, Ann Arbor, MI) allowing pre- and post-contrast imaging with the specimen in the same position to better facilitate secondary qMRI analysis. A baseline MRI series was acquired pre-contrast. Contrast solution was then manually injected by syringe and consisted of Gd-DTPA (Gadolinium-diethylenetriamine pentaacetic acid) mixed with normal saline (3:1 saline to Gd-DTPA). Total infusion per specimen was 61 ml (35 ml via SFA, 13 ml via ATA, and 13 ml via PTA). Static fat-suppressed and unsuppressed post-contrast T1-weighted three-dimensional gradient-echo images were generated for each MRI exam.

Fat-suppressed MRI images were utilized for qMRI analysis, to best visualize Gd-DTPA by eliminating normal bone marrow signal. Software customized by a study investigator (JPD) based on IDL 6.4 (Interactive Data Language, Exelis, Boulder, Colorado) allowed volumetric analysis. MRI examinations were reformatted to the sagittal-oblique plane to capture the entire length of the ACL and allow optimal segmentation for qMRI analysis.

**Fig. 1:**
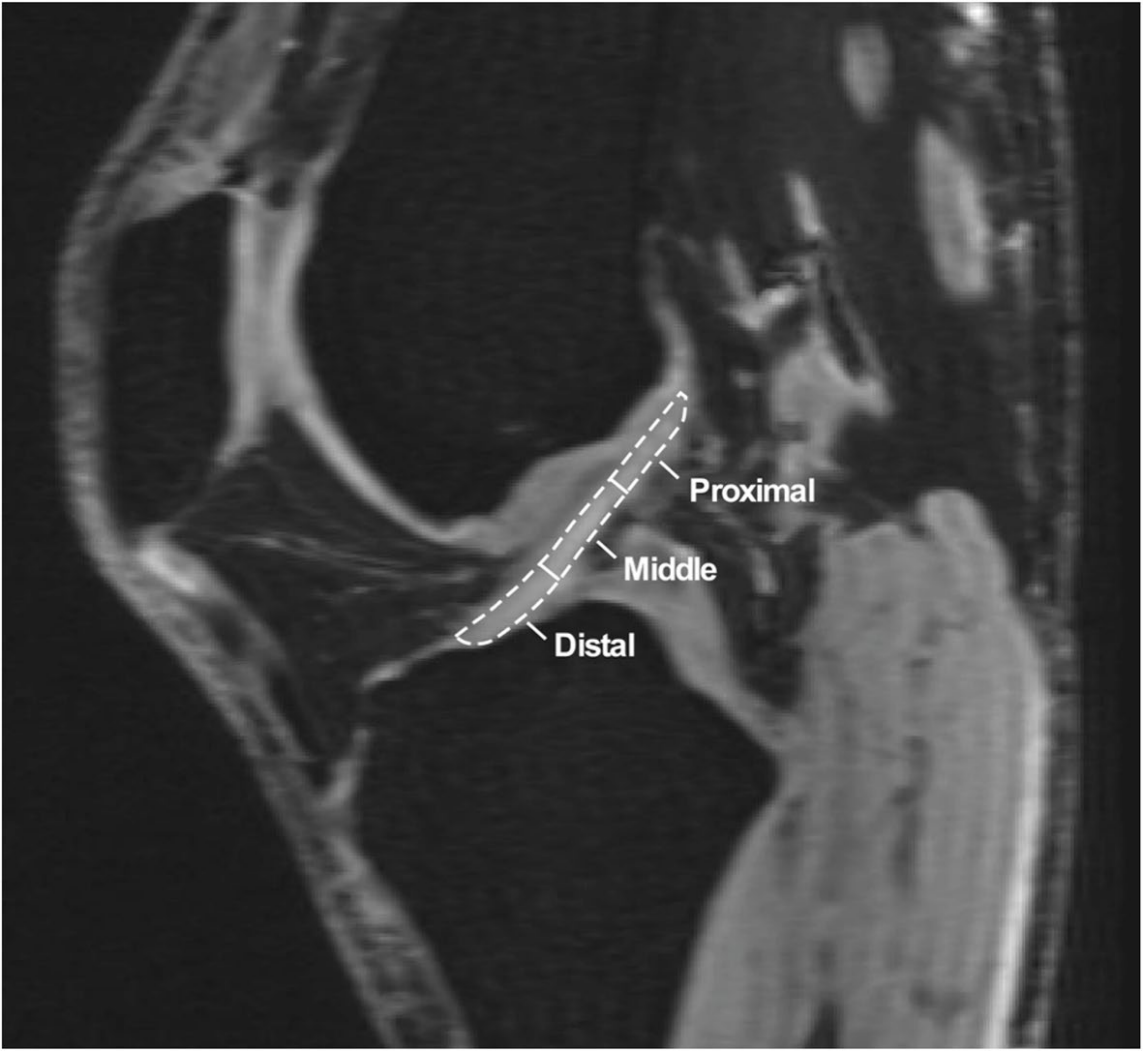
On the sagittal-oblique sections of the ACL, the substance of the ligament was divided into three regions of interest: proximal, middle and distal

**Fig. 2:**
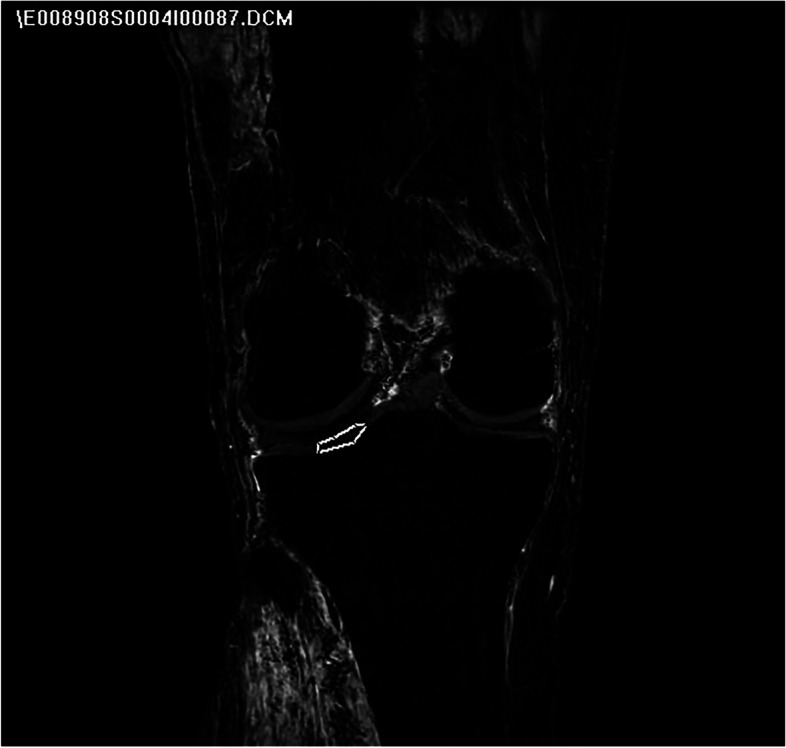
Signal intensity was normalized against non-enhancing tibial plateau articular cartilage using coronal plane images

On sagittal-oblique projections, the ACL was divided into three regions of interest (ROI): proximal, middle, and distal (Fig. 1). Entire ACL length on baseline MRI was divided into 3 equivalent length zones. Signal intensity change within each designated ROI from pre- to post-contrast MRI series were quantified on all slices in which the ACL could be visualized in its entirety from the femoral to the tibial insertion. Signal intensity was normalized against non-enhancing tibial plateau articular cartilage, with the ROI sampled at the widest region of tibial plateau cartilage (Fig. 2). Weighted means of signal intensity measurements per voxel were calculated producing a single measurement of signal intensity enhancement for each zone. Increase in signal intensity represented the relative increase in vascularity to any particular region. This technique has previously been described for assessment of relative arterial contributions in various anatomic sites, including for soft tissues of the knee [21, 23–28].

### Statistical analysis

Our biostatistics department was consulted for statistical analysis. Descriptive statistics (mean and standard deviation) were calculated for all zones. Mean perfusion was compared across zones using repeated measures ANOVA, and pairwise comparisons were performed using Bonferroni correction. A sample size calculation, to detect difference in relative vascularity between zones, was performed for analysis of a continuous endpoint using two samples. There was no previous data in the literature regarding quantitative relative vascularity of ACL zones to base calculations on. A threshold effect size of 20% (difference in relative perfusion) and standard deviation of 15% yielded a required sample size of 9 subjects per comparison group to detect a difference of 20% relative perfusion.

## Results

**Fig. 3:**
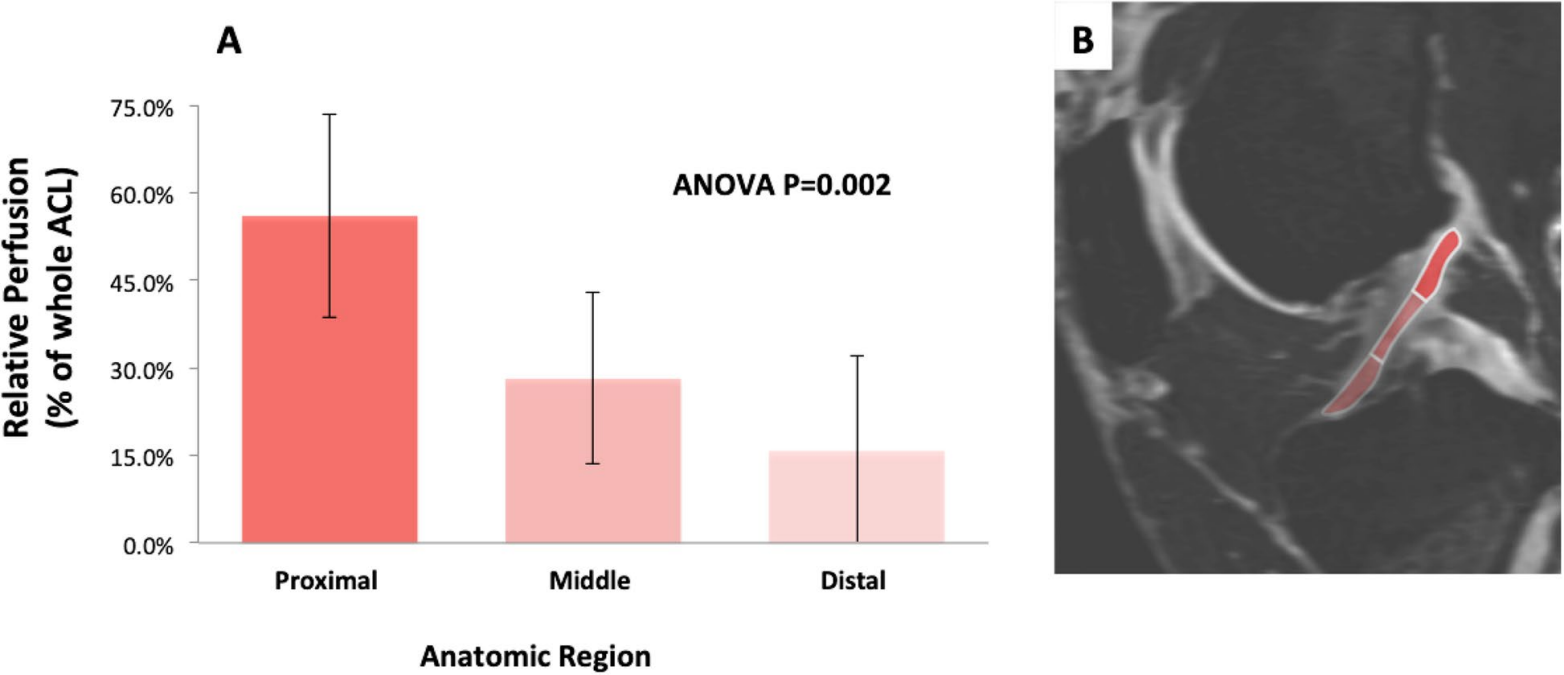
The greatest mean relative perfusion (as a percentage of the total ACL perfusion) was seen in the proximal ACL region (56% ± 17.4%), followed by the middle region (28.2% ± 14.6%), and the least relative perfusion was seen in the distal region (15.8% ± 16.3%)

All 14 specimens successfully underwent cannulation and qMRI. Greatest mean relative perfusion was found within the proximal ACL region (56.0% ± 17.4%); (with 56.0% of the total measured signal enhancement of the entire ACL found within the proximal aspect of the ACL), followed by the middle region (28.2% ± 14.6%), and the least relative perfusion in the distal region (15.8% ± 16.3%); (Fig. 3). Repeated measure ANOVA showed significant differences among zones (*P* = 0.002), and pairwise comparisons indicated perfusion to the proximal third was significantly greater than both middle (*P* = 0.007) and distal thirds (*P* = 0.001). Perfusion to the middle third was not significantly different from the distal third (*P* = 0.281). Descriptive and comparative statistics for differential regional ACL vascularity are presented in Table 1.

**Table 1:** Descriptive and Comparative Statistics for Regional ACL Vascularity

Descriptive Statistics			
Region	Average Perfusion^a^	Std. Dev	
Proximal	56.0%	17.4%	
Middle	28.2%	14.3%	
Distal	15.8%	21.4%	
**Comparative Statistics**			
**Comparison**	**Difference**^a^	**95% CI**	**P**
Proximal vs. Middle	27.9%	7.5% - 48.2%	**0.007**
Proximal vs. Distal	40.2%	18.0% - 62.5%	**0.001**
Middle vs. Distal	12.4%	−6.4% - 31.2%	**0.281**

^a^Percentage value represents percentage of total ACL vascularity

**Fig. 4:**
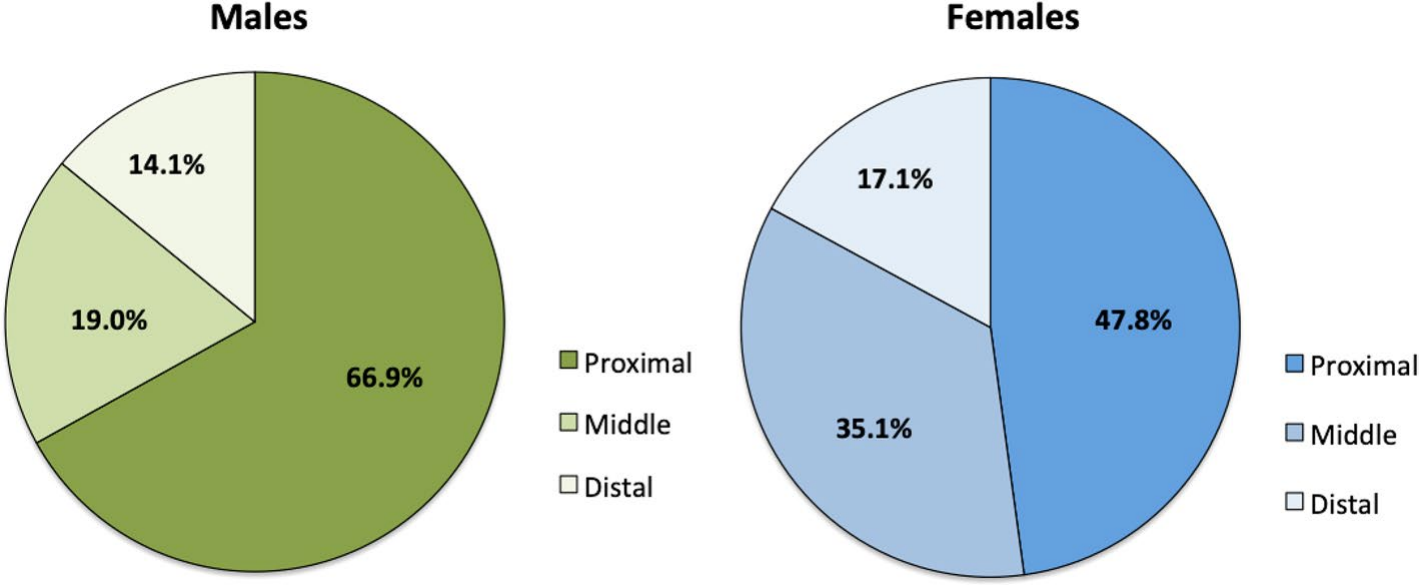
Comparison of regional ACL perfusion between genders revealed differences between males and females

Comparison of regional ACL perfusion between genders revealed differences (Fig. 4). Males were found to have a mean of 66.9% (SD 17.3%) of overall ACL vascularity within the proximal portion (with 66.9% of total measured signal enhancement from the entire ACL seen within the proximal third), 19.0% (SD 12.1%) within the middle portion, and 14.1% (SD 17.5%) within the distal portion. Females were found to have 47.8% (SD 13.0%) of overall ACL vascularity within the proximal portion, 35.1% (SD 12.9%) within the middle portion, and 17.1% (SD 16.4%) within the distal portion. Subgroup comparison of regional ACL perfusion between males and females using t-test showed relative vascularity to the proximal zone significantly greater in males (*P* = 0.036), with vascularity to the middle region significantly greater in females (*P* = 0.036), and no relative vascularity difference within the distal region (Table 2). It must be noted, however, that despite statistically significant findings, this comparison was not adequately powered.

**Table 2:** Summary Statistics for Regional ACL Vascularity by Gender

Region	Male^a^	Female^a^	Difference ^b^	95% CI	P
Proximal	66.9% (17.3%)	47.8% (13.0%)	19.1%	1.5% to 36.7%	**0.036**
Middle	19.0% (12.1%)	35.1% (12.9%)	−16.1%	−30.9% to − 1.3%	**0.036**
Distal	14.1% (17.5%)	17.1% (16.4%)	−3.02%	−22.9% to 16.9%	0.75

^a^Data are presented as mean (standard deviation). Percentage value represents percentage of total ACL vascularity

^b^Difference is calculated as Male value - Female value (positive represents greater value from Male group)

## Discussion

The study purpose was to quantitatively compare perfusion of different anatomic ACL regions using gadolinium-enhanced MRI. There were statistically significant differences in regional perfusion, with the proximal third receiving majority of total perfusion, followed by the middle and distal thirds. There was a statistically significant difference in regional perfusion between genders, with males demonstrating greater perfusion proximally, although this study was not initially powered for this post-hoc analysis.

To our knowledge, no prior studies quantitatively assessed human ACL vascularity in situ using noninvasive techniques used in the clinical setting. The qMRI findings corroborate historical qualitative studies that, using dissection, tissue clearing, and injection techniques, showed a broader vascular network of small capillaries in the proximal ACL. The vascular supply to the ligament substance derives from the synovium, rather than through the osseous insertion sites [15]. Prior evidence suggests the greatest concentration of vessels is near the femoral origin [17]. One prior study quantified vascularity to differential ACL zones in a canine model using intraarticular dissection and placement of probes to measure hydrogen washout [16].

The greater concentration of vessels and greater relative blood flow to the proximal third ACL suggest that this region may have greater healing potential, although it is important to note that vascularity is not the only factor involved in healing. This is important clinically in several aspects: (i) indicating patients for operative or conservative management, (ii) surgical technique with respect to the renewed interest in primary repair, and (iii) tissue healing following repair or reconstruction with or without remnant preservation. It is possible preservation of a stump or remnant of the native ACL insertion may provide improved biological healing potential, in the form of native collagen structure, matrix composition, or vascularity. From a practical standpoint, in the clinical setting remnant preservation is more typically practiced on the tibial side.

There is evidence suggesting some full thickness ACL tears in middle-age female skiers can be managed nonoperatively with good results [9]. It is possible that this is due to avulsion of the ACL from the femoral origin in this particular group; however, whether or not these tears even heal (or if they scar down to the PCL in a favorable position) is not known. Whether these results can be extrapolated to other demographic groups or other injury mechanisms remains unknown, as literature on nonoperative management of full-thickness ruptures is extremely limited [29, 30]. Regarding operative treatment, there is renewed interest in primary ACL repair in the setting of partial thickness tears [31] or femoral-sided avulsions [32]. Historically, open primary ACL repair was abandoned due to unacceptably high failure rates at mid-term follow-up [8]. With improved diagnostic capabilities [33] and arthroscopic repair approaches, contemporary rehabilitation protocols (early mobilization rather than casting and immobilization), and most importantly improved indications, results of selective ACL repair in recent literature have been generally encouraging [11, 34–36], although there are no high quality comparative studies with long-term follow-up [12, 13].

It is not known if regional differences in vascularity are re-established after ACL graft reconstruction. The reconstructed ACL graft differs in collagen microstructure, non-collagenous extracellular matrix composition, and material properties from the native ACL. The process of ACL graft revascularization is incompletely understood. Although patella bone-tendon-bone autografts heal at the graft-tunnel junction through creeping substitution at the bone-to-bone interface [37], it is unclear if distribution of vascularity returns. It is unknown how this process occurs with soft tissue grafts. The finding of increased proximal perfusion is perhaps most relevant to remnant-preserving reconstruction, which has recently become increasingly reported although there is no definitive high quality data supporting its use [38, 39]. Based on our findings, it could be hypothesized that femoral remnant preservation may provide more biologic benefit than tibial remnant preservation, at least from a vascularity standpoint. Although from a practical clinical standpoint it is typically preservation of the tibial remnant that is practiced. It is possible that some form of neovascular collateral circulation arises and perfuses the graft via the remnant stump; however, further study is required to elucidate when, how, or if this process occurs. Alternatively, the relatively lower proportional perfusion to the distal ACL may suggest that at least from a vascularity standpoint, tibial remnant preservation does not contribute much. From a tissue-healing standpoint, the presence of greater perfusion to the proximal ACL on qMRI supports the previously hypothesized preferential healing potential in the proximal region. At the very least, from a biological standpoint, increased vascularity would support fibrovascular scar formation, possibly facilitating a non-anatomic healing response of the ruptured femoral stump to nearby tissues. Nonetheless, it must be noted that perfusion alone does not equate to healing potential as numerous other factors are involved, including need for responding cells (either intrinsic or extrinsic cells that repopulate the graft) and the appropriate growth factors and other signaling molecules in the healing tissue.

Although post-hoc subgroup analysis of differential vascularity by gender was not powered in our study, gender differences are worthy of continued investigation, as female athletes are reported 2–10 times more likely than male athletes to suffer ACL injuries [40]. Anatomic factors, including ACL thickness, notch geometry, quadriceps angle, and tibial slope, that differ between genders have been hypothesized to impact ACL injury propensity [40–42]. While it has not been reported in the literature, it is also possible differences in vascularity may contribute to an observed discrepancy in injury rates, although further study certainly is required to draw this conclusion. Our findings support a recent report describing accumulated tissue fatigue damage in the femoral enthesis region of ACL tissue from patients with non-contact ACL failure [43].

There are important limitations to this study, which limit direct clinical relevance of the findings. First, although in situ ACL vascularity is assessed without intra-articular dissection, this is a cadaveric study and results should be cautiously extrapolated to living tissue. The ACL is a dynamic structure exposed to a complex mechanical loading environment, including cyclic compression, tension, and shear, and likely also undergoes physiologic aging, all of which likely affect tissue vascularity, while MRI in this study was done as a single timepoint static examination. ACL vascularity likely changes with age and injury (altering local microvascular environment), so any results from cadaveric imaging studies of intact ACLs must be cautiously extrapolated to ruptured ACLs in living patients in the clinical setting. In addition, the study sample was limited, and subgroup analysis by gender was not adequately powered. No group matching was done for the gender comparison. Furthermore, average donor age was 51.1 years, on the older side for patients seeking ACL injury consultation. This introduces sampling bias, as ACL vascularity in a 50-year-old knee may differ from that of a 30- or 15-year-old. Additionally, manual injection of MRI contrast solution may introduce variability to perfusion pressure which could have been better controlled through an MRI-safe pressure-controlled infusion pump. However, the same study investigator performed all contrast injections using the same syringe sizes and same technique to minimize variability. It is also possible specimen size relates to degree of MRI contrast solution uptake within the ACL; we did not calibrate contrast dose to specimen size, as in previous vascularity studies of the knee using this technique, all knees were injected with the same absolute volume of contrast. Additionally, this study did not specifically assess for reliability and reproducibility related to the ROI analysis which would have been beneficial. This methodology has been used in prior research and findings have been supported by both latex dissection and contrast Computed Tomography imaging. Finally, in order to improve clinical impact of this research, further work should investigate the role differential vascularity plays in tissue healing, in the setting of nonoperative management, primary repair, or reconstruction using various graft types. Future vascularity studies should also be done in post-operative knees, with comparison groups, long-term follow-up, and correlation to clinical outcomes, when possible.

## Conclusions

Relative perfusion to the in situ human ACL, assessed using qMRI in cadaveric knees, differs by anatomic zone, with greatest perfusion at the proximal third compared to middle and distal thirds. These findings are valuable for the field of ACL surgery, as differential perfusion carries implications for management of ACL injuries. Increased blood supply may signify greater biologic activity and thus possibly greater healing potential. In context of the literature that has identified a small cohort of patients who have good outcomes following nonoperative management of ACL ruptures, as well as the renewed interest in primary ACL repair, the finding of increased vascularity to the femoral region of the ACL may, in part, provide a physiologic basis. Differential vascularity, where present, may impact injury, healing, and clinical outcomes.

## Data Availability

The datasets used and/or analyzed during the current study are available from the corresponding author on reasonable request.
